# Five-year effect of community-based intervention Hartslag Limburg on quality of life: A longitudinal cohort study

**DOI:** 10.1186/1477-7525-9-11

**Published:** 2011-02-27

**Authors:** Saskia PJ Verkleij, Marcel C Adriaanse, WM Monique Verschuren, Eric C Ruland, Gerrie CW Wendel-Vos, Albertine J Schuit

**Affiliations:** 1 Department of Health Sciences VU University, Amsterdam, the Netherlands; 2 National Institute for Public Health and the Environment, Bilthoven, the Netherlands; 3 Netherlands Institute for Health Promotion and Disease Prevention, Woerden, the Netherlands

## Abstract

**Background:**

During the past decade, quality of life (QoL) has become an accepted measure of disease impact, therapeutic outcome, and evaluation of interventions. So far, very little is known about the effects of community-based interventions on people's QoL. Therefore, the effect of an integrative cardiovascular diseases community-based intervention programme 'Hartslag Limburg' on QoL after 5-years of intervention is studied.

**Methods:**

A longitudinal cohort study comparing 5-year mean change in QoL between the intervention (n = 2356) and reference group (n = 758). QoL outcomes were the physical and mental health composite scores (PCS and MCS) measured by the RAND-36. Analyses were stratified for gender and socio-economic status (SES).

**Results:**

After 5-years of intervention we found no difference in mean change in PCS and MCS between the intervention and reference group in both genders and low-SES. However, for the moderate/high SES intervention group, the scales social functioning (-3.6, 95% CI:-6.1 to -1.2), physical role limitations (-5.3, 95% CI:-9.6 to -1.0), general mental health (-3.0, 95% CI:-4.7 to -1.3), vitality (-3.2, 95% CI:-5.1 to -1.3), and MCS (-1.8, 95% CI:-2.9 to -0.6) significantly changed compared with the reference group. These differences were due to a slight decrease of QoL in the intervention group and an increase of QoL in the reference group.

**Conclusion:**

Hartslag Limburg has no beneficial effect on people's physical and mental QoL after 5-years of intervention. In fact, subjects in the intervention group with a moderate/high SES, show a decrease on their mental QoL compared with the reference group.

## Introduction

During the past decade there has been growing interest in measuring people's quality of life (QoL). Traditionally, outcome measurements in health care have mostly been determined by objective medical evaluation [1]. The interest in assessing QoL stems from recognition of the importance of patients' own perception of their health status and well-being. QoL has become an accepted measure of disease impact, therapeutic outcome, and evaluation of interventions.

Chronic diseases often affect people's QoL. Research shows that people with diabetes mellitus type 2, obesity, and cardiovascular diseases (CVD) have an decreased QoL [2-5]. Moreover, people with favourable levels of CVD risk factors have greater longevity and tend to have a better QoL [2]. Therefore, health promotion may not only stimulate a healthy lifestyle but may also improve people's QoL. A widely advocated strategy in public health is community-based health promotion.

In 1998, a community-based CVD prevention program was initiated in the Netherlands, in the Maastricht region of the province of Limburg. The goal of Hartslag Limburg, Dutch for Heartbeat Limburg, is to reduce the CVD risk by a reduction in fat intake, an increase in physical activity, and smoking cessation [6-9]. Hartslag Limburg and other community-based prevention programs have been proven effective in reducing cardiovascular and lifestyle risk factors [9-12]. However, until now the effects of community-based interventions on people's QoL are not known. This is striking because QoL is a clinically important outcome of people's perspective on well-being. Therefore, the aim of the present study was to investigate the effect of Hartslag Limburg on QoL after 5-years of intervention.

## Methods

### Hartslag Limburg

In 1998, the community-based intervention project Hartslag Limburg started. The aim of the project was to decrease the prevalence of CVD in the general population of the Maastricht region (population 185,000) by encouraging the inhabitants to become physically active, reduce their fat intake, and quit smoking. Hartslag Limburg, incorporated two strategies: 1) a population strategy aimed at all inhabitants and specifically at low SES groups, and 2) a high-risk strategy focusing on individuals diagnosed with CVD or multiple CVD risk factors (e.g. hypertension, cholesterol, and overweight) [9]. The main partners in the community project are the city councils of Maastricht and four adjacent municipalities, the Regional Public Health Institute Maastricht (RPHI), two community social work organizations, and the regional community healthcare organization. Collaboration among these partners is achieved through nine local health committees that organize activities which promote and facilitate healthy lifestyles. From 1999 until 2003, a total of 790 interventions have been implemented, of which 590 were major interventions (193 diet, 361 physical activity, and 9 antismoking). Almost 50% of the interventions took place in low-income areas. Examples of activities include computer-tailored nutrition education, nutrition education tours in supermarkets, public-private collaboration with the retail sector, television programs, food labeling, smokefree areas, creating walking and bicycling clubs, walking and cycling campaigns, and a stop-smoking campaign, in addition to commercials on local television and radio, newspaper articles, and pamphlet distribution. A more detailed description of the project is available elsewhere [8].

### Ethics approval

This study was approved on 18 August 1998 by the Dutch Medical Ethics Committee TNO. Chairman of committee: Dr. C.H.M. Kleemans. Letter of reference; CO/TW 2599/10049.

### Study population

In this study, a cohort design was used to investigate the effect of the intervention. Changes observed in the intervention group were compared with changes in a reference group. The study population of both intervention and reference area originated from two former monitoring studies conducted by the Dutch National Institute for Public Health and the Environment [13,14].

The source population of the intervention region consisted of 13,184 men and women. From this group a gender- and age-stratified sample of 4,500 subjects was selected. This was done because the aim was to include at total of 3,000 subjects in the baseline measurement. A response rate of at least 65% was anticipated based on previous experiences. Of the selected 4,500 sample, 441 men and women were excluded because they had moved to another region. The remaining 4,059 subjects were invited to participate. 3,232 (80%) whished to participate in the study, but for economical and logistical reasons we were forced to include 3,000 subjects only. So the remaining 232 subjects that reported their interest in the study were excluded after the 3000 was reached. Of these 3,000 subjects, 2,414 (81%) participated in the 5-year follow-up measurement in 2003. In order to standardize the difference in age range in the two populations, participants younger than 30 years were excluded (n = 58) from the intervention population. Therefore, we analysed the data of 2,356 subjects from the intervention region.

The source population in the reference region was smaller, and for this reason all subjects were included in the study. These subjects participated in an ongoing cohort (the Doetinchem cohort), in which all participants were physically examined in 1998 and 2003. In 1998, a total of 1,115 were invited, of which 895 subjects participated (80%). Of these 895 subjects, 758 subjects (85%) participated in the follow-up measurement in 2003.

In total, analyses were performed on a population of 3,114 (2,356 in the intervention region and 758 in the reference region) men and women aged 31 to 70 years. Participants from both the reference and intervention areas were informed that the aim of the study was to monitor change in risk factors in adults over a 5-year period. Thus, they were not aware of the underlying aim of the present study. The study population has been described in more detail elsewhere [9].

### Data Collection

The measurements performed in the intervention and reference group consisted of identical standardized methods. In the reference area, data collection started in January and lasted until December of the same year. In the intervention area, data collection started in August (same year as reference group) and lasted until February the next year. The measurements included a physical examination at the Regional Public Health Institute and a self-administered questionnaire. The staff that performed the physical examination in the intervention region was not blinded for the goal of the study, but they were unaware of the values of the pre-intervention measurement when conducting the post-intervention measurement. The self-administered questionnaire consisted of questions on demographics, health status, QoL, current smoking, physical activity, diet, and chronic diseases. During the physical examination, blood pressure (systolic and diastolic), height, weight, waist circumference, and total and HDL cholesterol concentration were measured.

#### Quality of life

QoL was measured by the Dutch version of the RAND-36 Health Survey (RAND-36) [15], which was translated from the standardized SF-36 Health Survey [16]. The RAND-36 consists of 36 questions which comprises of eight multi-item scales: physical functioning, social functioning, role limitations due to physical health problems, role limitations due to emotional problems, general mental health, vitality, bodily pain, and general health perception. In addition, two summary scores representing physical (PCS) and mental health (MCS) are generated. All scales were scored from 0 to 100, with higher scores indicating a better QoL [17]. The RAND-36 is a validated, reliable, and responsive measure with good psychometric properties [18]. The RAND-36 comprises of the same items as the SF-36 [19], however, the methodology to derive the final scores is different, but the effect on the final score is minimal [16]. It is suggested that a minimum of three to five points difference on any given scale may be considered clinically important [20].

#### Risk factors and diseases

Socio-economic status (SES) was defined by the highest level of education that was completed. Education was measured on a nine-point ordinal scale ranging from elementary school to completed university. Low socio-economic status was defined as lower vocational or primary school. Current smoking status was assessed by asking the respondents whether they had smoked the last seven day (yes/no). Participants that indicated that they did smoke were categorized as smoker. Body mass index (BMI) was calculated as weight divided by height squared (kilograms per square meter). In this calculation one kilogram was subtracted from the measured weight, in order to adjust for the light indoor clothing. Presence of diseases at baseline was based on self-reported prevalence of one of the following diseases: myocardial infarction, stroke, cancer, or diabetes mellitus type 2. The occurrence of diseases between baseline and follow-up is determined by the absence of a disease at baseline and the self-reported presence of one or more of the above mentioned diseases at follow-up.

### Statistical analysis

Descriptive data (means, standard deviation, and percentage) of the baseline characteristics of the intervention and reference population were presented for men and women separately. First, differences in study sample characteristics of the intervention and reference population by sex were examined using Students t-test for continuous variables and χ^2^-tests for categorical variables. Next, the effect of Hartslag Limburg on QoL was investigated by comparing change in the two summary RAND-36 scores, PCS and MCS, between the intervention group and the reference group using regression analyses. The dependent variable is change in PCS and MCS. Group status (intervention/reference) is the independent variable. The analyses were performed separately for men and women and adjusted for age, SES, presence of chronic diseases at baseline, occurrence of chronic diseases between baseline and follow-up, and mean of baseline and follow-up measurement of the variable under study. This last adjustment was done to neutralize possible effects of regression to the mean [21]. Finally, since Hartslag Limburg has a specific focus on low-SES groups, additional regression analyses were also stratified for SES. For all statistical testing, we used two-sided hypothesis testing with an alpha level of < 0.05. Data were analysed using SAS software version 9.1.

## Results

### Study population

Baseline characteristics of men and women measured in 1998 for the intervention and the reference population who completed follow-up in 2003 are shown in Table 1.

**Table 1:** Baseline characteristics stratified by sex of intervention and reference population (1998) who completed follow-up in 2003

	Men		Women	
		
	Intervention (n = 1187)	Reference (n = 349)	Intervention (n = 1169)	Reference (n = 409)
**Demographics**				
Age (years)	50.6 (9.8)*	52.2 (9.9)	50.6 (9.7)	51.3 (10.4)
Low socio-economic status (%)	44.6	43	60.7	61.3
Current smoking (%)	23.9	24.7	26.7	22
BMI^a ^overweight^b ^(%)	64	60	48	51
**Diseases (self reported)**				
Myocardial infarction (%)	3.1	3.4	0.7	0.7
Stroke (%)	0.8	0.6	0.6	1.0
Cancer (%)	2*	4	4.2	5.6
Diabetes mellitus (%)	2.9	1.4	1.5	2.2
**Quality of Life**				
Physical functioning (PF)	89.2 (15.5)	89.7 (16.0)	85.0 (17.7)	85.9 (18.6)
Social functioning (SF)	88.6 (19.0)	90.2 (16.4)	84.7 (21.0)	82.6 (21.9)
Role limitations physical (RP)	86.8 (28.6)	86.9 (27.2)	80.2 (35.2)	78.9 (34.7)
Role limitations emotional (RE)	89.4 (26.8)	91.3 (22.7)	85.4 (31.9)	85.4 (31.1)
General mental health (MH)	78.7 (15.6)	80.0 (13.5)	73.3 (16.9)	74.3 (15.1)
Vitality (VT)	68.9 (17.6)*	70.9 (15.8)	63.5 (18.3)	63.5 (16.9)
Bodily pain (BP)	84.7 (21.0)	86.7 (18.2)	79.5 (23.4)	78.2 (21.5)
General health perception (GH)	69.5 (17.2)*	72.7 (15.9)	68.1 (18.1)	69.4 (17.2)
MCS^a^	51.3 (9.0)	52.2 (7.9)	49.1 (10.0)	49.0 (9.5)
PCS^a^	51.0 (7.4)	51.5 (7.4)	49.6 (8.9)	49.5 (9.1)

*Difference between intervention and reference group (*p < 0.05) (bolded)*. Data presented as mean (SD) or as percentage.

^a ^BMI, Body mass index; MCS, Mental Health Composite score of RAND-36; PCS, Physical Health Composite score of RAND-36.

^b ^Overweight was defined as body mass index of ≥ 25 kg/m^2^.

Mean age of both populations was approximately 51 years. There were no significant differences in baseline characteristics in women between the two populations. However, men in the intervention group were younger, scored significantly lower on prevalence of cancer, vitality, and general health perception than men in the reference group. Additional analysis showed that at follow-up, responders (n = 3,114) (the total number of subjects with a pre- and post intervention measurement (intervention n = 2,356 and control n = 758)) compared with non-responders (n = 682) scored higher on baseline PCS (50.4 vs. 49.0) and MCS (50.3 vs. 48.9), whereas no differences were found regarding age, gender, and SES.

### Effect on QoL after 5-years

The mean and adjusted difference in change in QoL among men and women in the intervention and the reference group after 5-years of intervention are presented in Table 2. After 5-years of intervention we found no difference in mean change in both PCS and MCS between the intervention and reference group across gender. For women, the differences between intervention and reference group were significant on the subscales social functioning (mean change between intervention and reference group -4.3, 95% CI: -6.9 to -1.7), vitality (-3.0, 95% CI: -4.9 to -1.1), and bodily pain (-2.8, 95% CI: -5.5 to -0.2). For men there were no significant differences between the intervention and the reference group on any of the eight subscales, nor on the summary PCS and MCS scales.

**Table 2:** Mean change in QoL^a ^by sex after 5-years of intervention

Quality of Life	Men			Women		
		
	Intervention	Reference	**Adj difference **^**b **^**(95% CI)**^**c**^	Intervention	Reference	**Adj difference **^**b **^**(95% CI)**^**c**^
PF^a^	-0.6	-1.7	0.7 (-1.0 to 2.4)	-1.6	-2.3	0.7 (-1.1 to 2.5)
SF^a^	-0.9	-0.1	-0.9 (-3.3 to 1.5)	-0.9	3.4	-4.3* (-6.9 -1.7)
RP^a^	-2.0	0.3	-2.7 (-6.7 to 1.3)	-3.7	0.4	-4.1 (-8.7 to 0.6)
RE^a^	-1.0	0.3	-1.2 (-5.0 to 2.6)	-0.4	0.0	-0.3 (-4.7 to 4.0)
MH^a^	-0.5	1.0	-1.5 (-3.1 to 0.2)	0.0	1.5	-1.6 (-3.3 to 0.2)
VT^a^	-0.4	0.0	-0.5 (-2.4 to 1.5)	-0.5	2.5	-3.0* (-4.9 to -1.1)
BP^a^	-1.6	-3.6	1.7 (-0.8 to 4.3)	-2.4	0.4	-2.8* (-5.5 to -0.2)
GH^a^	-2.6	-3.7	0.9 (-0.9 to 2.6)	-2.8	-2.3	-0.8 (-2.5 to 1.0)
MCS^a^	-0.2	0.7	-0.8 (-1.8 to 0.3)	0.2	1.3	-1.1 (-2.3 to 0.1)
PCS^a^	-0.7	-1.3	0.5 (-0.4 to 1.4)	-1.2	-0.7	-0.6 (-1.5 to 0.4)

*Difference between intervention and reference group (*p < 0.05) (bolded)*.

^a ^QoL, quality of life; PF, physical functioning; SF, social functioning; RP, role limitations physical; RE, role limitations emotional; MH, general mental health; VT, vitality; BP, bodily pain; GH, General health perception; MCS, Mental Health Composite score of RAND-36; PCS, Physical Health Composite score of RAND-36. between baseline and follow-up, and the mean of baseline and follow-up of the variable under study.

^b ^Adjusted difference in change between the intervention and the reference group for age, level of education, presence of self reported diseases (myocardial infarction, stroke, cancer, diabetes mellitus) at baseline (1998), occurrence of diseases (myocardial infarction, stroke, cancer, diabetes mellitus)

^c ^95% CI, 95% confidence interval.

### Social economic status

The mean change and adjusted difference in change in QoL among low and moderate-high SES groups in the intervention and reference group after 5-years of intervention are presented in Table 3. In the low SES intervention group (n = 1,239), physical functioning decreased significantly less (1.9, 95% CI: 0.0 to 3.8) during follow-up compared with the reference group (n = 401). For the moderate or high SES intervention group (n = 1,117), the scales social functioning (-3.6, 95% CI: -6.1 to -1.2), physical role limitations (-5.3, 95% CI: -9.6 to -1.0), general mental health (-3.0, 95% CI: -4.7 to -1.3), vitality (-3.2, 95% CI: -5.1 to -1.3), and MCS (-1.8, 95% CI: -2.9 to -0.6) significantly changed compared with the reference group (n = 357). These differences were due to a slight decrease of QoL in the intervention group compared with a slight increase of QoL in the reference group.

**Table 3:** Mean change in QoL among low and moderate-high SES^a ^groups after 5-years of intervention

QoL	**Low SES**^**a**^					**Moderate or high SES**^**a**^				
	Baseline **I**^**a**^ (n = 1239)	Baseline **R**^**a**^ (n = 401)	Mean change **I**^**a**^	Mean change **R**^**a**^	**Adj. diff**^**b**^ **(95% CI)**^**c**^	Baseline **I**^**a**^ (n = 1117)	Baseline **R**^**a**^ (n = 357)	Mean change **I**^**a**^	Mean change **R**^**a**^	**Adj diff**^**b**^ **(95% CI)**^**c**^
PF^a^	83.8 (18.6)	85 (20.1)	-0.9	-3.1	1.9* (0.0 to 3.8)	90.9 (13.4)	90.6 (13.7)	-1.2	-0.8	-0.7 (-2.3 to 0.8)
SF^a^	84.8 (21.6)	84.2 (21.2)	0.2	1.9	-1.9 (-4.4 to 0.6)	88.9 (18)	88.2 (18.1)	-2.1	1.6	-3.6* (-6.1 to -1.2)
RF^a^	80.7 (34)	81.1 (34.1)	-2.2	-0.9	-1.8 (-6.2 to 2.6)	86.9 (29.6)	84.3 (28.8)	-3.4	1.7	-5.3* (-9.6 to -1.0)
RE^a^	86 (31)	87.9 (28.1)	0.0	-1.4	1.3 (-2.8 to 5.3)	89.2 (27.3)	88.4 (27.2)	-1.6	1.8	-3.2 (-7.4 to 0.9)
MH^a^	74.2 (17.4)	75.3 (15.8)	0.2	0.4	-0.2 (-2.0 to 1.5)	78.2 (14.8)	78.7 (13.1)	-0.8	2.3	-3.0* (-4.7 to -1.3)
VT^a^	64.7 (18.8)	65.6 (17.1)	-0.2	0.3	-0.6 (-2.5 to 1.4)	68 (17.2)	68.4 (16.4)	-0.7	2.4	-3.2* (-5.1 to -1.3)
BP^a^	79.5 (23.7)	79.5 (22.6)	-1.8	-1.1	-0.7 (-3.4 to 1.9)	85.1 (20.3)	85 (17.4)	-2.3	-1.8	-0.6 (-3.1 to 2.0)
GH^a^	66.8 (18.1)*	68.9 (17.9)	-2.8	-3.1	0.0 (-1.7 to 1.8)	71.2 (16.8)*	73.2 (15)	-2.7	-2.8	0.0 (-1.8 to 1.8)
MCS^a^	49.7 (10.1)	50.0 (9.2)	0.4	0.6	-0.2 (-1.3 to 0.9)	50.9 (8.9)	50.9 (8.7)	-0.4	1.5	-1.8* (-2.9 to -0.6)
PCS^a^	49.2 (8.7)	49.4 (9.1)	-0.9	-1.0	0.0 (-0.9 to 1.0)	51.7 (7.4)	51.6 (7.3)	-1.0	-0.9	-0.2 (-1.1 to 0.7)

*Difference between intervention and reference group (*p < 0.05) (bolded)*.

^a ^SES, socio-economic status; QoL, quality of life; I, Intervention region; R, Reference region; PF, physical functioning; SF, social functioning; RP, role limitations physical; RE, role limitations emotional; MH, general mental health; VT, vitality; BP, bodily pain; GH, General health perception; MCS, Mental Health Composite score of RAND-36; PCS, Physical Health Composite score of RAND-36.

^b ^Adjusted difference in change between the intervention and the reference group for age, gender, presence of diseases (myocardial infarction, stroke, cancer, diabetes mellitus) at baseline (1998), occurrence of diseases (myocardial infarction, stroke, cancer, diabetes mellitus) and between baseline and follow-up, and the mean of baseline and follow-up of the variable under study.

^c ^95% CI, 95% confidence interval.

## Discussion

This study focused on exploring the effect of a community-based prevention program on people's QoL. This is, to our best knowledge, the first study that prospectively determined the effect of a CVD community-based intervention (Hartslag Limburg) on people's QoL. We concluded that Hartslag Limburg has no beneficial effect on people's physical and mental QoL after 5-years of intervention. Only for women, differences between intervention and reference group were significant for the subscales social functioning, vitality, and bodily pain. In fact, subjects in the moderate/high SES intervention group, show a decrease on their mental health composite score compared with the reference group. These differences were due to a slight decrease of QoL subscales social functioning, general mental health and vitality in the moderate/high SES intervention group and an increase of those three QoL subscales in the reference group.

Several outcomes of the effects of the program Hartslag Limburg have already been reported. Hartslag Limburg was not effective in changing smoking behaviour [22], but was effective in reducing other cardiovascular and lifestyle risk factors (e.g. BMI, blood pressure, energy intake, and time spent on walking) [9,12]. In this study, we anticipated a small decrease in the QoL in both groups (due to ageing), being less pronounced in the intervention group. However, this was not observed. On the contrary, the present study found a non-significant tendency for a reduction in QoL in the intervention group, and an improvement of QoL in the control group (six of the eight scales for women and four of the eight scales for men). Apparently the beneficial changes in CVD risk factors associated with the intervention program did not translate into a better perceived QoL. Maybe the cardiovascular and lifestyle risk changes were too modest to influence people's QoL. Seasonality can not explain the outcome of the study, because the pre- and post intervention measurement of subjects in intervention and control group took place in the same month.

Research has shown that SES is associated with (self-rated) health status [23]. Since Hartslag Limburg has a specific focus on low SES groups, analyses were stratified for SES. Previous analyses showed that Hartslag Limburg beneficially affected BMI, waist circumference, blood pressure, energy intake, fat intake, walking, and bicycling in low SES groups [9,12]. Hence, we particularly anticipated an effect on QoL in this group. However, except for physical functioning no effects were observed in the low SES group.

Community-based CVD prevention programs are a widely advocated strategy in public health. So far, no studies have reported on the effects of community-based interventions on QoL. There is also limited data on the effect of health promotion programs and QoL. Comparison of outcomes is difficult because of differences in time periods over which the effects were measured, used methods, interventions, and study populations. Yet, there are some related studies that put our results in perspective. Improvement in the mental component of QoL has been reported after a cardiovascular lifestyle modification program of one year [24]. Also Lobo et al. found less impairment in QoL in the intervention group compared with a control group after an intervention program of 21 months [25]. The only study that also did not report a beneficial effect of a lifestyle program on QoL is the study of Cupples & McKnight, who investigated the effect of a 2-year health promotion program five years after enrolment in patients with angina [26]. However, these studies all focused on patients at high cardiovascular risk and were based on individually targeted interventions.

The strengths of our study are the longitudinal design, the use of a reference group, a large sample of subjects, and a follow-up of 5 years. The large number of participants included in this study ensures enough power to detect small differences. Finally, we used the RAND-36, which is a validated, reliable, and responsive questionnaire to measure QoL [18].

This study also has some limitations that should be addressed. First, the number and selectiveness of drop-outs may have biased the results. In our study, responders scored higher on baseline PCS and MCS compared with non-responders at the follow-up. No differences between non-responders and responders were found in age, gender, and SES. In this study however, over 80% of the subjects completed both the baseline and the 5-year follow-up measurement. So, it is not likely that drop-out might have changed our results. Second, it is well known that presence of chronic diseases can negatively effect people's QoL [3-5]. Therefore, the results of our study were adjusted for the presence or occurrence of myocardial infarction, stroke, cancer, and/or diabetes mellitus type 2. Unfortunately, no information was available about all chronic diseases (e.g. chronic obstructive pulmonary disease (COPD), depression and inflammatory bowel diseases). So, we could not control for them. However, the percentage of people in our study population, who are suffering from COPD and/or inflammatory bowel diseases, would probably be low. So, it is not likely to influence our results to a great extent.

In summary, this study showed that five years of community-based prevention did not lead to an improvement in QoL. In fact, subjects in the intervention group with a moderate/high SES, show a decrease on their mental QoL compared with the reference group. Although the health effects of Hartslag Limburg and other community based intervention have been previously established, this study does not provide an indication that these types of programs should be implemented to favourably improve the QoL in the general population.

## Conclusion

We found that Hartslag Limburg has no beneficial effect on people's physical and mental QoL after 5-years of intervention. No substantial effects were observed in men and women. However, people in the intervention group with a moderate or high SES had a relative decrease in mental QoL compared to their peers in the reference group.

## Competing interests

The authors declare that they have no competing interests.

## Authors' contributions

SPJV prepared the article and performed the data-analyses. MCA contributed to writing the article. AJS was project leader of Hartslag Limburg and contributed to writing the article. GCWW performed the project coordination of Hartslag Limburg and contributed to writing the article. ECR conceived the study and was project leader. WMMV was project leader of the Doetinchem cohort. All authors have read and approved the final version of the article.
